# Analysis of Biological Images and Quantitative Monitoring Using Deep Learning and Computer Vision

**DOI:** 10.3390/jimaging12020088

**Published:** 2026-02-18

**Authors:** Aaron Gálvez-Salido, Francisca Robles, Rodrigo J. Gonçalves, Roberto de la Herrán, Carmelo Ruiz Rejón, Rafael Navajas-Pérez

**Affiliations:** 1 Departamento de Biología y Geología, Área de Genética, Universidad de Almería, 04120 Almería, Spain; ags408@ual.es; 2 Departamento de Genética, Universidad de Granada, 18071 Granada, Spain; frobles@ugr.es (F.R.); rherran@ugr.es (R.d.l.H.); carmelo@ugr.es (C.R.R.); 3 Departamento de Ecología, Universidad de Granada, 18071 Granada, Spain; rogo@ugr.es; 4 Research Unit Modeling Nature (MNat), Universidad de Granada, 18071 Granada, Spain

**Keywords:** deep learning, computer vision, automated counting, biological imaging

## Abstract

Automated biological counting is essential for scaling wildlife monitoring and biodiversity assessments, as manual processing currently limits analytical effort and scalability. This review evaluates the integration of deep learning and computer vision across diverse acquisition platforms, including camera traps, unmanned aerial vehicles (UAVs), and remote sensing. Methodological paradigms ranging from Convolutional Neural Networks (CNNs) and one-stage detectors like You Only Look Once (YOLO) to recent transformer-based architectures and hybrid models are examined. The literature shows that these methods consistently achieve high accuracy—often exceeding 95%—across various taxa, including insect pests, aquatic organisms, terrestrial vegetation, and forest ecosystems. However, persistent challenges such as object occlusion, cryptic species differentiation, and the scarcity of high-quality, labeled datasets continue to hinder fully automated workflows. We conclude that while automated counting has fundamentally increased data throughput, future advancements must focus on enhancing model generalization through self-supervised learning and improved data augmentation techniques. These developments are critical for transitioning from experimental models to robust, operational tools for global ecological monitoring and conservation efforts.

## 1. Methodological Approach

**Figure 1:**
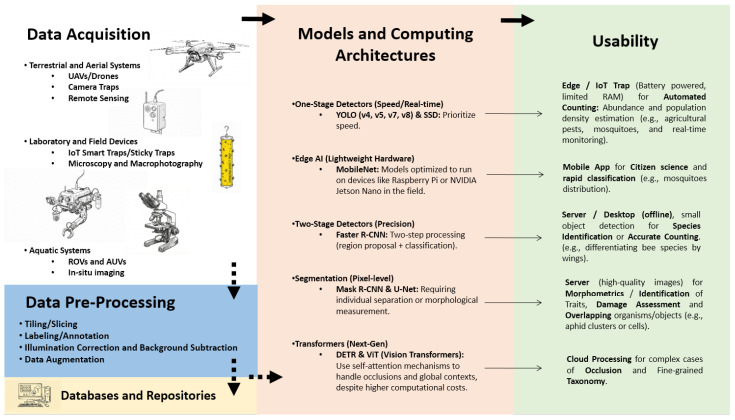
Schematic overview of the data management workflow. The figure details the computational infrastructure implemented for the systematic organization, long-term storage, and automated processing of environmental monitoring datasets.

Figure 1 illustrates the methodological pipeline for automating the management and analysis of biological images. Key stages include data acquisition, data pre-processing, selection of models/architecture, and use cases (usability). Some common methods and tools are outlined below.

### 1.1. Data Acquisition

One of the most used methods to obtain wildlife images is the motion-activated camera, commonly referred to as ‘camera trap’. These provide a non-invasive, discreet, autonomous, and cost-effective approach for collecting large volumes of image data in wildlife monitoring studies [1]. This methodology has been increasingly adopted across a wide range of taxa and environments, including terrestrial, freshwater, and marine ecosystems [2]. Despite their widespread use, a major limitation of camera trap surveys is the post-acquisition time- and labor-intensive manual processing of images, which is required to extract biologically meaningful information. This constraint significantly increases analytical effort and limits the scalability of camera trap studies at large spatial and temporal extents [3].

To address this challenge, automated image classification based on machine learning techniques has become a key component of modern camera trap workflows. In particular, deep convolutional neural networks (DNNs) have demonstrated strong performance in automated feature extraction and image classification tasks, achieving high accuracy while enabling the processing of millions of images in relatively short computational times. These capabilities make DNN-based approaches especially suitable for large-scale camera trap datasets [4,5,6].

Another common method is the use of unmanned aerial vehicles (UAVs), commonly referred to as drones. This enables the acquisition of high-resolution aerial imagery through programmable flight paths, allowing the coverage of large spatial areas while posing minimal risk to researchers. UAV platforms can be equipped with a wide range of sensors, including conventional RGB cameras, thermal infrared cameras, multispectral sensors, and LiDAR systems, which has substantially expanded their applicability in ecological research.

In particular, drone-based approaches have proven effective for the study and monitoring of large vertebrates, primarily mammals and birds [7,8]. Thermal cameras mounted on UAVs enable the efficient discrimination of endothermic organisms from a thermally cooler background, facilitating detection even in densely vegetated environments or under low-light conditions [9,10]. In addition, drones have been widely applied across a range of ecological studies, including abundance estimation, habitat mapping, and environmental impact assessment [11,12].

Nevertheless, although drone-based surveys represent an efficient and cost-effective method for wildlife monitoring, it is essential to consider that operational factors such as flight altitude, approach distance, and UAV-generated noise may elicit stress responses and behavioral alterations in terrestrial, aerial, and aquatic animals. These potential impacts highlight the importance of incorporating animal welfare considerations and implementing ethically sound and carefully designed sampling protocols when employing drones in ecological research [13].

Not only drones, but remote sensing in general (i.e., including satellite imagery, see below) has become a key tool for biodiversity studies, as it enables the simultaneous observation of spatial, temporal, and structural patterns within ecosystems or large areas. Its applications encompass both plant and animal biodiversity, either directly or through the characterization of habitats and their dynamics [14].

High spatial resolution refers to the ability to capture images in which each pixel represents a relatively small area on the ground (generally <10 m), allowing detailed analysis of landscape structure and habitat distribution. This resolution is critical for assessing both plant and animal biodiversity: in vegetation studies, it facilitates the identification of patches, edges, and degrees of fragmentation [15], while in wildlife studies, it is used to analyze habitat connectivity and, in some cases, to directly detect large animals in open ecosystems. These applications are particularly relevant for the conservation and monitoring of threatened species and the planning of protected areas [16].

Hyperspectral remote sensing employs sensors capable of capturing hundreds of narrow and contiguous spectral bands, so that each pixel contains detailed information on reflectance across a wide range of wavelengths. This technique provides precise data on the biophysical and biochemical properties of vegetation, enabling differentiation of species, plant communities, and physiological states. Such information is essential for evaluating plant diversity and ecosystem health [17], and it also serves as a critical foundation for animal biodiversity studies [18], since vegetation composition and quality directly influence the availability of resources, shelter, and ecological niches for fauna.

Thermal remote sensing is based on measuring infrared radiation emitted by terrestrial or aquatic surfaces, allowing for the estimation of surface temperature, which influences the distribution and behavior of many species. These data are used, for instance, to assess vegetation thermal stress, monitor wildfire dynamics [19], or evaluate the thermal suitability of habitats for animal species [20]. Moreover, thermal sensors have proven useful for the direct detection of endothermic animals, particularly under nocturnal or low-visibility conditions.

Small satellite constellations, composed of multiple observation platforms (typically nanosatellites or microsatellites) operating in low Earth orbit, offer high temporal revisit frequency. This capability allows for the monitoring of rapid changes in vegetation cover and condition, associated with phenological processes or environmental disturbances [21], as well as evaluating how these changes affect animal biodiversity [22]. The availability of frequent imagery facilitates early detection of habitat degradation and the assessment of its impacts on biological communities.

The Light Detection and Ranging (LiDAR) system is an active remote sensing technique that uses laser pulses to measure distances and generate highly detailed three-dimensional models of the terrestrial surface and vegetation. LiDAR data allow for precise estimation of structural landscape and forest variables, including vegetation density, biomass, and habitat complexity, which are key indicators of functional diversity [23]. These structural variables are closely linked to animal biodiversity [24], as they determine the availability of shelter, nesting sites, and resources, and are widely used to model species richness and abundance [25]. Its ability to provide three-dimensional information significantly complements and enhances analyses based on passive imagery. A comprehensive summary of current methodologies is provided in Table 1. 

### 1.2. Image Processing Methodologies

The evolution of automated counting in biological imaging has transitioned from manual feature engineering to robust deep learning frameworks. These methodologies can be categorized by their architectural design (Convolutional Neural Networks CNNs or Vision Transformers ViTs) and the detection paradigms they use.

Convolutional neural networks continue to serve as the primary architecture for biological object detection due to their strong inductive bias toward capturing local spatial structures.

#### 1.2.1. Two-Stage Object Detection

These detection paradigms fundamentally rely on deep convolutional neural network (CNN) architectures to prioritize detection accuracy. Subsequent iterations such as Faster R-CNN use a CNN backbone to process the entire image and extract hierarchical feature maps that encode rich semantic information. These detectors operate structurally in two distinct steps. First, a proposal mechanism (e.g., a Region Proposal Network) generates a sparse set of candidate regions from the feature maps. Second, a specialized head pools and processes each region for classification and bounding-box refinement [26,27]. The original R-CNN architecture laid the foundation for this paradigm. However, the evolution toward sharing full-image feature computation (Fast R-CNN and Faster R-CNN) significantly reduced computational costs while maintaining the high accuracy of the two-stage design [28,29]. A significant development in this framework was the introduction of Mask R-CNN [30]. Mask R-CNN expanded the Faster R-CNN architecture by incorporating a third parallel branch for pixel-level instance segmentation. This enhancement enables simultaneous object detection and segmentation. This capability is essential for applications that require detailed visual understanding.

#### 1.2.2. One-Stage Object Detection

This alternative approach treats detection as a single, dense prediction problem. The network predicts object class scores and bounding box offsets directly on a regular grid in a single pass. Representative one-stage methods include Single Shot Multibox Detector (SSD) and You Only Look Once (YOLO), which prioritize simplicity and speed by eliminating the region proposal step and performing detection in a single pass [31,32]. Historically, one-stage models were much faster, but they often exhibited lower accuracy than two-stage models. This difference was primarily due to an extreme imbalance between the foreground and background classes during training. Innovations such as focal loss (RetinaNet) addressed this issue by allowing one-stage detectors to match or exceed the accuracy of two-stage detectors while maintaining single-pass speed [33].

#### 1.2.3. Transformer-Based Architectures

Recent advances in computer vision have introduced transformer-based architectures as a powerful alternative to purely convolutional models. While this type of architecture is widely used for natural language processing models, its application to image recognition remains limited [34]. Subsequent developments have consolidated this technology as a dominant framework for complex image recognition, including the identification of biological specimens in heterogeneous environments and video images [35].

Among these architectures, hierarchical models like the Swin Transformer (Swin-T) have emerged as powerful alternatives to standard CNNs. By limiting self-attention to local windows, Swin-T reduces computational complexity compared to the original ViT while maintaining superior accuracy for dense object detection, although it remains too computationally intensive for most battery-operated edge devices. Unlike CNNs, which rely on local receptive fields, Transformers employ self-attention mechanisms that explicitly model long-range dependencies across the entire image [35]. This property is particularly beneficial for automated counting in biological imagery, where individuals may be small, partially occluded, or distributed in dense and irregular spatial patterns.

The Detection Transformer (DETR) is one of the most prominent applications of this paradigm in object detection. It introduces an end-to-end detection framework that combines a convolutional backbone with a transformer encoder–decoder. This framework reformulates detection as a direct set prediction problem and eliminates hand-designed components, such as anchor boxes and non-maximum suppression [36]. In the context of biological counting, DETR’s set prediction approach is particularly transformative. Traditional methods often rely on density map estimation, which can result in the loss of individual-level information in dense clusters. However, by treating counting as a direct set prediction task, transformer-based models can effectively disambiguate overlapping organisms or cells. This allows them to avoid the post-processing artifacts typically introduced by nonmaximum suppression (NMS) in dense scenarios [37]. Despite their advantages, transformer-based approaches introduce higher computational costs and increased model complexity. Consequently, many practical systems for automated biological counting use hybrid architectures that balance the efficiency of convolutional operations with the global reasoning capabilities of self-attention mechanisms.

Although Transformer-based models like DETR excel in disambiguating overlapping instances and capturing global context, this capability comes at a significant computational cost. Recent comparative benchmarks indicate that while Transformers often achieve higher Average Precision (AP) in complex, occluded scenes, their inference speed is considerably slower than state-of-the-art one-stage detectors. For instance, experimental evaluations in surveillance and agricultural contexts show that while DETR-based architectures can outperform YOLO models in detection accuracy for dense clusters, they may require significantly more inference time per image, making them less viable for real-time applications on standard edge devices. Therefore, for resource-constrained scenarios such as battery-powered smart traps or onboard UAV processing, lightweight CNNs (e.g., YOLOv8-Nano, MobileNet) remain the preferred choice.

The application of deep learning in the field of ecology has undergone rapid and substantial expansion. However, extant reviews have been predominantly fragmented, with a focus on either specific taxonomic groups or particular acquisition platforms. For instance, recent comprehensive reviews have exclusively addressed insect monitoring [38], mammal detection via camera traps [39], or marine life identification [40]. Similarly, other surveys have limited their scope to specific technologies, such as UAVs for wildlife tracking [41] or acoustic monitoring [42]. While a number of broader reviews are available, they frequently prioritize the algorithmic architecture over the biological application [43] or focus exclusively on the ‘counting’ problem without addressing the integration of heterogeneous data sources [44]. This review addresses a significant lacuna in the extant literature by offering a holistic, multi-taxa and multi-platform perspective. In contrast to previous studies, we have synthesized automated counting methodologies across the three major data acquisition pillars—ground-level (camera traps/IoT), low-altitude (UAVs), and orbital scales (satellite imagery)—and across diverse biological kingdoms. This cross-cutting approach facilitates the identification of unifying challenges, such as the scarcity of ground truth data and domain adaptation, which remain obscured when analyzing each domain in isolation.

### 1.3. Model Efficiency and Deployment Considerations

Deploying deep learning models on edge devices, such as smart camera traps or UAVs, requires striking a balance between detection accuracy and computational efficiency. Large-scale architectures, including deep convolutional neural networks and transformer-based models such as ViTs and DETR, have demonstrated superior performance in complex visual scenarios, such as occlusions and cluttered backgrounds. However, their high parameter count, memory footprint, and computational demands often limit their applicability to resource-constrained hardware platforms [4,27,34,36,37].

In practical field deployments, particularly those requiring real-time inference and low energy consumption, lightweight object detection models are preferred. Architectures such as SSD and YOLO-based detectors are explicitly designed for real-time performance and have become the dominant choice for edge applications thanks to their favorable accuracy–speed trade-off [31,32]. Recent advances have produced even lighter variants, including optimized YOLOv8 architectures, which allow for efficient deployment on embedded systems while maintaining competitive detection performance [27,45].

Several model optimization strategies are commonly used to bridge the performance gap between high-capacity models and edge-compatible architectures. Techniques such as model pruning, quantization, and knowledge distillation reduce computational complexity and memory usage by eliminating redundant parameters, lowering numerical precision, and transferring knowledge from larger networks to smaller ones. These approaches are particularly valuable for ecological and agricultural monitoring applications, which require autonomous operation, limited power availability, and low-latency decision-making.

The combination of lightweight detection architectures and model optimization techniques extends battery life and reduces inference latency in autonomous monitoring systems without substantially compromising detection accuracy. This makes the systems well-suited for long-term deployment in real-world ecological surveillance scenarios.

In Table 2, a comparative analysis between architectures is shown.

### 1.4. Resources: Image Databases for Training Models, Platforms and Monitoring Systems

Table 3 synthesizes the primary datasets available for insect recognition, detailing the specific taxa groups, image counts, and the source or main characteristics of each resource to facilitate quick comparison.

Based on the summarized resources, a distinct trend is observable towards high-volume datasets for economically significant orders, specifically Lepidoptera and Coleoptera. The data highlights a strategic shift in the field from static, museum-based imagery to dynamic, non-lethal data collection methods involving automated camera traps (e.g., AMI Consortium). This transition facilitates the training of robust AI models capable of real-time pest detection and biodiversity monitoring in complex, natural environments, moving beyond controlled laboratory settings.

## 2. Applications

### 2.1. Automated Image-Processing Methods for Pest Control

Automated image-processing for pest detection has demonstrated significant utility across a wide variety of insect taxa, covering diverse ecological niches ranging from flying pests and leaf-miners to soil and ground pests. These methods utilize various acquisition modalities, including traps, sticky cards, greenhouse cameras, and UAVs. Current research indicates successful application across at least five major insect orders: Coleoptera, Lepidoptera, Diptera, Orthoptera, and Hemiptera (Table 4). However, the field faces distinct limitations. Small insects like thrips or mites often require macro lenses to avoid appearing as sensor noise [46]. Cryptic species pose another challenge, as identical visual appearances may require DNA analysis or microscopic examination for differentiation, a task current image recognition cannot perfectly solve. Furthermore, occlusion—where insects hide under leaves or overlap—remains a significant barrier to accurate counting and identification [47].

#### 2.1.1. Diptera: Mosquitoes and Flies

Automated image processing for Diptera is one of the most developed areas in insect computer vision, primarily due to the significant public health and agricultural risks these insects pose. Recognition often focuses on leg length, wing structure, and even wingbeat frequency (if video/audio is combined with imagery). A combination of yellow sticky-paper traps and/or LED illumination is used in greenhouse settings or orchards [67]. Multiple DL models (Faster R-CNN, SSD, RetinaNet, YOLOv5) are used for detection. Research is split between species-level identification (often via wing analysis) and real-time surveillance (using smart traps and IoT).

- Mosquitoes (Culicidae) and Disease Surveillance

Mosquito (Culicidae) research is highly focused on distinguishing between medically important genera (*Aedes*, *Anopheles*, *Culex*), even identifying their gender. Advanced systems have been deployed to monitor these vectors. For instance, Dragonfly Robot utilizes YOLOv4 to detect and map mosquito hotspots in real-time, while the Mosquito Alert project leverages citizen science and deep learning to identify invasive species like *Aedes albopictus*. VisText-Mosquito is a cutting-edge multimodal dataset that integrates visual data (images) with natural language reasoning to analyze mosquito breeding sites. IoT Smart Trap includes a smart trap system embedded with neural networks that processes mosquito images locally to save bandwidth and power.

Methodologically, ensemble models have proven effective. Goodwin et al. [48] utilized a multitiered CNN ensemble to distinguish known from unknown species, a critical feature for monitoring invasives. Real-time field deployment has also advanced, with Kittichai et al. [49] developing embedded systems for high-accuracy identification. Other studies have achieved 97–98% accuracy using architectures such as VGG-16, ResNet-50, and YOLOv514 [50].

- Flies (Tephritidae & Muscidae) and Agricultural Pests

Research in flies often focuses on the olive fruit fly and other tephritids that cause massive crop damage. See, for example, Remote Fruit Fly Detection by Molina-Rotger et al. [51], which combines traditional Machine Learning (Random Forest, SVM) with IoT devices (Raspberry Pi) for in situ monitoring of olive flies, reaching 94.5% accuracy.

Perre et al. [68] published a foundational study using wing morphometrics and landmark detection to automate the identification of fruit flies. Also, focusing on wing spots, landmarks, and edge geometries, Guerrón & Rocha [52] achieved 99% accuracy at the genus level for 48 different species. A continuous 40-day monitoring experiment in two greenhouses (cherry tomato and strawberry) showed average recognition accuracy ~96%, even with tiny pests and complex backgrounds. The monitored pests included tobacco whiteflies, fruit flies, and houseflies, but also thrips, leaf miners and aphids [69]. Another study showed that DL detection on trap images is feasible even in orchard setups with complex backgrounds; different detectors were compared, showing the practicality of multi-class pest detection beyond just a single species [70]. In a recent paper, FlydAI (FlyDetector AI), a tool trained to detect different *Drosophila melanogaster* morphs was developed [71].

#### 2.1.2. Hemiptera: Sap-Sucking Insects

Processing images of Hemiptera (sap-sucking insects) is particularly complex due to the insect’s minute size, high polymorphism, and tendency to aggregate in dense clusters. Despite these challenges, detection is vital for managing pests like aphids, whiteflies, psyllids, and planthoppers, which are major vectors for plant viruses. Thus, field or laboratory leaf images and sticky traps are used to collect data. Detection is generally performed via convolutional neural networks (CNNs) or segmentation pipelines.

- Aphids and Small Sap-Suckers (Aphididae & Aleyrodidae)

These insects are often detected using smart traps or high-resolution field imagery. A recent study collected thousands of sorghum-field images and used state-of-the-art detectors (VFNet, GFLV2, PAA, ATSS) on annotated aphid-cluster patches. Results showed stable average precision and recall, and performance improved by merging nearby clusters and removing tiny noise clusters [46]. Huang et al. [53] introduced the BEAM (Bi-Enhanced Attention Mechanism) to help YOLOv8 focus on tiny targets, achieving 95.4% accuracy in real-field conditions. Masuko & Kikuta [54] modified a YOLOv8 model to classify nymphs and adults of *Acyrthosiphon pisum* with 95.9% precision, despite their minute differences and variable lighting. An application used specifically to overcome the lack of training data for whiteflies has been developed, improving detection rates in greenhouse environments where small insects are hard to photograph. CNNs or traditional image processing are also used to count aphids on leaves, achieving accuracies up to ~96% [72]. In these cases, systems based on semantic segmentation and object detection were able to accurately identify aphid clusters [73].

- Hemiptera (‘true bugs’) and Heteroptera (Miridae and Pentatomidae)

These larger Hemipterans are often identified using overall body photographs obtained from museum collections or via citizen science. Popkov et al. [74] demonstrated that CNNs could identify plant bugs with high accuracy, even in groups that usually require genital dissection for confirmation. Popkov et al. [75] created The Eurygaster Project, a dedicated tool for identifying Sunn pests (due to highly polymorphic true bugs) in wheat fields, using a microservice architecture to support field identification for farmers.

- Psyllids and Planthoppers (Psyllidae and Delphacidae)

Research in this group of organisms often integrates multimodal imaging to handle occlusion by leaves. Tang et al. [47] combined RGB, Near-Infrared (NIR), and Thermal imaging to detect Psyllids hidden by leaf shading, achieving a 95.3% counting accuracy. Cen et al. [45] targeted small planthoppers using random splicing methods to enhance the training dataset for improved early-warning alerts.

#### 2.1.3. Lepidoptera: Butterflies and Moths

Research in the automated identification and monitoring of Lepidoptera has accelerated rapidly due to advances in Convolutional Neural Networks (CNNs) and deep learning architectures like YOLO and EfficientNet. Individuals from Lepidoptera are often identified by wing patterns and color. Monitoring often involves specialized hardware (specialized moth light traps use AI to track nocturnal species) and mobile software (electronic/pheromone traps for butterflies). The AMI Data Platform is the industry standard for processing large-scale moth trap imagery. The AMMOD Project (Automated Multisensor Stations for Monitoring of Biodiversity) specifically deals with moth scanners that automate species identification using deep learning pipelines in field stations. The ButterflyCount App (European Butterfly Monitoring Scheme—eBMS) utilizes the Observation.org AI algorithm to provide real-time identification for citizen scientists across Europe.

The literature discusses the fundamental AI architectures used to classify Lepidoptera species based on wing morphology and pattern recognition. Ding & Taylor [76] developed one of the earlier foundational works using deep learning for real-time pest monitoring in agricultural traps. Syamsudin et al. [77] used EfficientNet-B0 to classify 25 species with 97.9% accuracy, highlighting the effectiveness of lightweight but deep architectures. Bjerge et al. [69] described a portable hardware system (AMT) that tracks, counts, and identifies live moths using a customized CNN. Yadav [56] compared ResNet50, EfficientNetB0, and a custom ButterflyNet model for successfully identifying 100 different species. He & Liu [78] explored the use of YOLOv7 to detect and classify Lepidoptera species in complex, noisy natural environments. A recent implementation in maize crops deployed a network of smart traps (IoT + camera + DL) that achieved 97% detection accuracy over multiple trap captures, enabling generation of infestation maps [57].

#### 2.1.4. Coleoptera: Beetles and Weevils

Automated image processing for Coleoptera is a rapidly growing field, particularly for agricultural pest management and biodiversity monitoring. Unlike Lepidoptera, which are often identified by flat wing surfaces, research on Coleoptera frequently employs 3D-aware processing and X-ray imaging to handle their rigid and hard wing covers (elytra), complex bodies, and internal infestations (especially in weevils).

Most studies focus on identifying diverse beetle families and genera, often using museum specimens or field camera traps. Valan et al. [58] achieved 96% accuracy in identifying 14 families of Coleoptera (including Curculionidae and Carabidae) using transfer learning with CNNs. Shah et al. [79] developed the DeepNet-9 model specifically for tiger beetles (Carabidae: Cicindelinae), achieving >90% accuracy using a modified SqueezeNet architecture. Liu et al. [59] used a three-stage pipeline utilizing Grounding DINO (for detection) and Mask2Former (for segmentation) to process large-scale beetle data from laboratory trays with 97.8% accuracy.

- Weevil Detection (Curculionidae) and Internal Infestation

Weevils represent a specific challenge because their larvae often develop inside seeds or trees, requiring non-visual sensing like X-ray or acoustics. Barboza da Silva et al. [60] used X-ray imagery processed with MobileNetV2 to detect maize weevils inside kernels where they are invisible to standard cameras. Kagan et al. [80] combined Google Street View data with deep learning to map urban infestations of *Rhynchophorus ferrugineus* at a city-wide scale. Al-Saqer & Hassan [81] proposed a specific Rostrum Analysis (snout detection) combined with Zernike moments to identify weevils from other insects with 97% accuracy.

- Scarabs and Leaf Beetles Detection: Agricultural Pest Monitoring

Karakan [61] utilized UAV (drone) imagery and YOLO architectures to detect *Leptinotarsa decemlineata* directly in the field with 99.8% training accuracy. Caruso et al. [82], using the entomoscope (a low-cost photomicroscope), were able to identify invasive emerald ash borers and other jewel beetles.

#### 2.1.5. Hymenoptera: Bees, Ants, and Wasps

Automated image processing for Hymenoptera is a critical area of research, as these insects serve as essential pollinators, predators, and biodiversity indicators. Detection is particularly challenging due to their small size, rapid movement, and the subtle morphological differences between species (e.g., wing venation). There are some field monitoring systems already developed. BioMONITec is a pollinator-focused project using YOLOv8 and macro-time-lapse camera traps in the Austrian Alps to monitor floral visitors (bees/wasps) with order-level classification. Also, the Julius Kühn-Institut created a DIY Camera Trap; an open-source, solar-powered intelligent camera trap designed to monitor flower-visiting bees and hoverflies in real-time.

- Bees (Apidae, Halictidae, and others)

Bee research is primarily focused on automated pollinator monitoring in the field and beehive health assessment [83]. Spiesman et al. [62] used EfficientNetV2L to achieve 98.1% accuracy using wing images. This study highlights that wing venation is a more stable feature for AI than whole-body images, which are affected by matted hair and body pose. Srinivas et al. [63] benchmarked several CNNs on field-captured images, finding MobileNet-V2 to be the most efficient, with 98.4% accuracy. Ratnayake et al. [84] focused on sub-species level identification (e.g., *Apis mellifera* vs. *Apis cerana*) with 94% accuracy. Karthiga et al. [85] explored multi-task learning to simultaneously identify the species and detect diseases or parasites like *Varroa mites*.

- Ants (Formicidae)

Ant research focuses on foraging behavior and density estimation in complex, crowded environments. Das et al. [64] utilized YOLOv8 to count ants in high-density scenes. It addresses the small object problem by slicing large images into smaller patches for processing, achieving ~88% precision. Silva et al. [86] developed a system for semi-quantitative density estimation. It uses MobileNet and EfficientNet V2-B0 as backbones to generate heatmaps of ant activity over time. Serra Montes [65] focused on multi-object tracking (MOT) to follow individual ants during foraging, improving the “Higher Order Tracking Accuracy” (HOTA) in overlapping scenarios.

- Wasps (Vespidae and Parasitoids)

Wasp research is divided between pest monitoring (wasps at beehive entrances) and taxonomy of tiny parasitic wasps. In Shirali et al. [66], tiny wasps (1.2–4.5 mm) were photographed and classified using Grounding DINO for automated cropping and specialized CNNs for identification. Achieved 96% accuracy at the genus level. DiversityScanner developed by Wührl et al. [87] is an automated lab-based device that uses CNNs to sort bulk samples of insects, including Hymenoptera, identifying them down to the family level with up to 100% accuracy. Chen et al. [88] developed WaspBase. Although primarily genomic, it provides the morphological ground truth (SEM images) often used to train high-resolution visual models.

#### 2.1.6. Key Limitations and Challenges

Despite the success of deep learning models, significant technical bottlenecks persist, particularly regarding small object detection. Insects like thrips or mites often occupy only a few pixels in an image, lacking sufficient spatial information for feature extraction. Standard architectures like YOLOv8 or DETR often fail in these scenarios because the down-sampling operations in their backbones result in the loss of fine-grained details necessary for detecting millimeter-sized targets. To address this, recent research suggests integrating Feature Pyramid Networks (FPNs), which fuse high-resolution features from shallower layers with semantic information from deeper layers, improving the detectability of small organisms. However, any model/algorithm optimization will still encounter the hard limit imposed by the acquisition method (i.e., low magnification).

Occlusion and dense aggregation present another major barrier. In scenarios where insects overlap or hide under foliage, standard non-maximum suppression (NMS) algorithms may inadvertently suppress valid detections. Transformer-based models (e.g., DETR) show promising results by treating detection as a set prediction problem, yet they struggle with the high computational cost of attention mechanisms in high-resolution images. Cryptic species differentiation remains a challenge where visual similarity exceeds the discriminatory power of RGB sensors, necessitating the integration of multimodal data (e.g., thermal or hyperspectral imaging) or DNA validation. Finally, the detection of invasive species may be controversial and case-dependent, since models may not be able to robustly detect new organisms for which they have not enough training data

#### 2.1.7. Architectural Selection: Trade-Offs in Insect Computer Vision

- The Case for CNNs: Constraints, Edge Computing, and Real-Time Surveillance

As highlighted for the case of Diptera (Mosquitoes) and Coleoptera (Agricultural Pests), the dominant architectures currently in use are CNN-based (specifically single-stage detectors like YOLOv4/v5/v8 and lightweight feature extractors like MobileNet). These architectures are mainly preferred due to:

Inductive Bias for Local Features: Insects often share distinct local morphological features (e.g., the rostrum analysis mentioned in weevils [79] or wing spots in fruit flies [52]). CNNs excel at capturing these local spatial hierarchies efficiently.

Edge Deployment (IoT): The text references “IoT Smart Traps” for mosquitoes and “embedded systems” [49]. A researcher should choose a CNN (like MobileNetV2 or YOLO) over a Transformer when the model must run locally on a Raspberry Pi or microcontroller to save bandwidth and battery. Transformers generally lack the throughput efficiency required for the real-time detection mentioned in the Dragonfly Robot or drone-based beetle detection [61].

Data Efficiency: Insect datasets are often small or highly imbalanced (e.g., invasive species). CNNs generally converge faster with less data compared to Transformers, which typically require massive datasets to learn global relationships.

- The Role of Attention and Transformers: Occlusion, Camouflage, and Fine-Grained Taxonomy

While standard CNNs dominate detection, the bibliography indicates a shift toward attention mechanisms for complex problems, such as Hemiptera (Sap-suckers), which aggregate in clusters, or Hymenoptera, requiring sub-species identification.

Handling Occlusion and Crowding: This review notes the difficulty of detecting Psyllids hidden by leaf shading [47] or counting ants in high-density scenes [83]. Here, architectures with attention mechanisms (like the BEAM mechanism mentioned for YOLOv8 [53] or full Vision Transformers) are superior. They allow the model to weigh the importance of different image parts globally, helping to distinguish an insect from a complex, noisy background where local convolution might fail.

Fine-Grained Classification: For tasks requiring differentiation between morphologically similar species (e.g., distinguishing *Apis mellifera* from *Apis cerana* [81] or recognizing specific wing venation in bees [62]), the global context provided by Transformers can outperform CNNs, provided computational resources are available (e.g., lab-based processing servers like the DiversityScanner [87], rather than field traps).

### 2.2. Image Processing in Aquatic Environments

**Figure 2:**
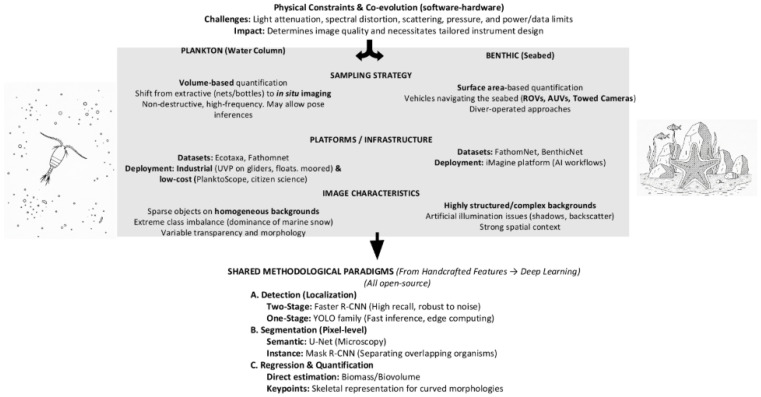
Schematic overview of aquatic imaging pipelines contrasting pelagic (plankton) and benthic domains. The diagram illustrates the co-evolution of hardware and software necessitated by physical constraints such as light attenuation and scattering. It highlights the distinct sampling strategies and image characteristics for water column versus seabed environments, converging into shared deep learning paradigms for detection (e.g., YOLO, Faster R-CNN), segmentation (e.g., U-Net), and regression tasks.

Unlike terrestrial or aerial environments, image acquisition in aquatic ecosystems requires dedicated instrument designs and tailored image-processing strategies (Figure 2). Light attenuation and spectral distortion with depth, strong scattering by suspended particles, and platform-dependent constraints (pressure, power consumption, data transmission) impose limitations that directly influence both image quality and downstream automated analysis. As a result, aquatic image-based research has co-evolved with advances in quantitative imaging instrumentation and machine learning-based processing pipelines, particularly for applications related to counting and characterizing biological objects.

Image-based approaches in aquatic environments predominantly target two ecological compartments: (i) the water column, where organisms and particles are suspended (plankton and marine snow), and (ii) the benthic environment, where organisms live attached to or in close association with the seabed. These two habitats differ fundamentally in object density, background complexity, illumination geometry, and sampling strategy, which in turn has led to partially distinct methodological developments.

#### 2.2.1. Plankton and Particles Imaging in the Water Column

- *From Net-Based Sampling to In Situ Imaging*

Historically, plankton abundance and community composition were quantified using nets, bottles, and pumps, followed by manual sorting and counting. While these extractive approaches remain indispensable for detailed taxonomic and physiological analyses, they integrate over large spatial and temporal scales, damage fragile organisms, and impose severe constraints on throughput. Comparative assessments have shown that net-based sampling is poorly suited to resolving fine-scale plankton patchiness and fragile taxa, motivating a transition toward non-destructive optical imaging systems deployed directly in the water column [89,90].

Quantitative in situ imaging instruments enable direct observation of plankton and particles within a controlled optical geometry, allowing object size, morphology, and concentration to be estimated from relatively small imaged volumes. Such systems form the observational backbone of many modern plankton ecology studies and generate image datasets that are well suited for automated analysis [91]. Early ship-based deployments paved the way for global observations, while recent developments emphasize miniaturization, automation, and long-term operation on autonomous platforms.

- *Imaging systems and platforms*

Among the most widely deployed in situ systems is the Underwater Vision Profiler (UVP) family, which images plankton and particles against a dark background using synchronized illumination. The most recent generation, UVP6, was explicitly designed for deployment on autonomous and cabled platforms, including profiling floats, gliders, and moorings, while maintaining quantitative consistency with earlier ship-based instruments [90]. UVP systems enable robust detection and sizing of particles and organisms typically larger than ~100 µm and have produced globally comparable datasets of plankton and marine snow (e.g., [91]).

Parallel developments have focused on affordability and accessibility. The PlanktoScope exemplifies this trend as an open-source, modular, and low-cost imaging platform suitable for laboratory analyses, nearshore deployments, and citizen-science applications [92]. While such systems may lack the environmental robustness of industrial-grade sensors, they substantially lower barriers to image-based plankton research and facilitate rapid methodological development.

Autonomous platforms increasingly carry imaging sensors, enabling observations at spatial and temporal scales unattainable with ship-based sampling alone. However, constraints on power consumption, onboard storage, and data transmission directly influence achievable image resolution and acquisition frequency, reinforcing the need for efficient and automated image-processing pipelines [90].

- *Image characteristics and preprocessing*

In situ plankton images typically contain sparse objects on relatively homogeneous backgrounds but exhibit extreme variability in size, transparency, and morphology. Marine snow and detrital particles often dominate numerically, leading to pronounced class imbalance that complicates automated identification and counting [91]. Consequently, preprocessing steps such as illumination correction, background subtraction, and threshold-based segmentation remain common, particularly for UVP-derived imagery [90].

While early pipelines relied heavily on fixed thresholds and handcrafted segmentation rules, these approaches are sensitive to variations in optical conditions and water properties. This limitation has driven a transition toward machine learning-based pipelines capable of adapting to heterogeneous image characteristics.

- *Automated identification and counting: methodological paradigms*

The transition from manual plankton counting to automated analysis mirrors broader developments in computer vision, evolving from classical machine-learning pipelines based on handcrafted features to end-to-end deep learning architectures. Contemporary approaches are commonly classified into detection-based, segmentation-based, and regression-based methodologies, depending on how objects are localized and quantified.

Detection-based methodologies localize individual organisms using bounding boxes and derive counts directly from detected instances. Two architectural paradigms dominate this category. Two-stage detectors, such as Faster R-CNN, first generate candidate object regions using a Region Proposal Network (RPN) before performing classification. These architectures are generally more robust to background noise and class imbalance and have been successfully applied to plankton and marine organism detection in microscopy and holographic imagery [93]. Their high recall makes them particularly suitable for monitoring rare or harmful taxa. One-stage detectors, including the YOLO family, perform localization and classification in a single step, offering substantially faster inference speeds. These models are attractive for near-real-time applications and have been explored in controlled monitoring environments, although they may exhibit increased sensitivity to complex backgrounds compared to two-stage approaches. Recent studies demonstrate that optimized detection models can be deployed on low-power edge devices, enabling on-site plankton monitoring in resource-constrained environments such as aquaculture systems. Such deployments highlight the growing relevance of model efficiency and hardware-aware optimization for operational plankton imaging.

Segmentation-based approaches assign pixel-level labels and are essential when organisms overlap or when precise morphological measurements are required. Semantic segmentation methods assign all pixels of a class to a single label, while instance segmentation explicitly differentiates between individual organisms. Instance segmentation architectures such as Mask R-CNN have proven more effective than semantic segmenters in separating individual specimens in crowded samples, including copepod aggregations or chain-forming diatoms. In parallel, U-Net and its variants remain widely used for microscopy-based plankton segmentation due to their efficiency and accuracy in image-to-image translation tasks. Advanced extensions, such as Regression U-Net (RU-Net) and A-Unet, have been developed for holographic microscopy, enabling simultaneous segmentation and estimation of three-dimensional position or dry mass from reconstructed volumes [93,94].

Regression- and keypoint-based quantification. Beyond explicit detection and segmentation, regression-based approaches directly estimate quantitative properties from images. CNN-based regression models have been used to predict organism size, biovolume, or biomass directly, bypassing intermediate segmentation steps [95]. Keypoint-based methods identify anatomical landmarks to construct a skeletal representation of an organism. This strategy is particularly valuable for taxa with curved or flexible morphologies, such as appendicularians or shrimps, where straight-line measurements are inadequate. By summing distances between keypoints, such approaches substantially reduce discrepancies relative to manual measurements and enable robust length estimation in complex morphologies.

Contemporary research increasingly emphasizes optimization, generalization, and scientific interpretability of deep learning models.

Hybrid architectures employing feature fusion combine local texture information with global shape descriptors to improve classification and counting performance, while model optimization techniques reduce computational overhead without sacrificing accuracy. At the same time, self-supervised learning (SSL) has emerged as a powerful strategy to address the scarcity of labeled plankton images. In-domain SSL approaches, trained directly on large unlabeled plankton datasets, have been shown to outperform traditional out-of-domain pre-training for specific classification tasks [96].

- *Beyond taxonomy: trait-based analysis*

Recent work emphasizes that plankton images encode rich functional and morphological information beyond taxonomic identity. Quantitative imaging enables extraction of individual-level traits such as size, opacity, shape complexity, pigmentation, and feeding-related features [97]. Trait-based approaches provide a continuous and ecologically meaningful description of plankton communities, capturing intra- and interspecific variability that is lost in purely taxonomic analyses.

Machine learning techniques have been adapted to extract such traits either through dedicated regression models or by reusing features learned during classification (95, 98]. In parallel, holographic microscopy combined with deep learning has enabled unprecedented tracking of individual microplankton throughout their life cycles, including direct measurements of growth, feeding, and life-history transitions [94]. These developments illustrate how image-based deep learning can link organism-level traits to ecosystem-scale processes.

#### 2.2.2. Image-Based Analysis of Benthic Communities

- *Imaging strategies and data characteristics*

Benthic imaging targets organisms living on or near the seabed, including corals, sponges, macroalgae, seagrasses, and benthic invertebrates. Image acquisition is typically conducted using diver-operated cameras, towed camera systems, remotely operated vehicles (ROVs), or autonomous underwater vehicles (AUVs). In contrast to plankton imaging [98], benthic imagery is characterized by highly structured and heterogeneous backgrounds, strong spatial context, and relatively stable object positions over short temporal scales [91,99].

Illumination in benthic environments is predominantly provided by artificial light sources mounted on the imaging platform. This introduces non-uniform lighting, shadows, backscatter, and color distortions that vary with camera altitude, viewing angle, and substrate complexity. These effects, combined with turbidity and uneven seafloor topography, substantially complicate automated segmentation and classification compared to pelagic imaging [99,100].

- *Datasets, platforms, and infrastructure*

As in plankton research, progress in automated benthic image analysis is tightly coupled to the availability of large, well-annotated datasets. Global initiatives such as FathomNet provide cross-platform image repositories with standardized annotations spanning pelagic and benthic environments, facilitating transfer learning and benchmarking across imaging systems [101]. Complementing this effort, BenthicNet specifically targets seafloor imagery and offers a curated, global compilation of benthic images designed to support deep-learning applications in habitat mapping and biodiversity assessment [102].

At the infrastructure level, platforms such as iMagine integrate annotation tools, model training pipelines, and deployment services for aquatic imaging applications, including benthic still images and video. These environments support reproducible workflows and facilitate the transition from experimental deep-learning models to operational monitoring tools [103]. Interactive machine-learning systems further enable human-in-the-loop annotation and validation, reducing expert workload while maintaining scientific reliability [104].

#### 2.2.3. Synthesis and Outlook

Across both planktonic and benthic habitats, image-based analysis has become an indispensable tool for aquatic environmental research. The transition from manual counting to automated, machine-learning–based pipelines has enabled orders-of-magnitude increases in data throughput and opened new avenues for quantitative ecological inquiry.

Despite rapid methodological advances, automated benthic image analysis remains less standardized than plankton imaging. Persistent challenges include strong class imbalance, high morphological variability, partial occlusion, and the high cost of expert annotation. Current research increasingly emphasizes transfer learning, data augmentation, active learning, and human-in-the-loop approaches to mitigate these limitations, closely paralleling trends observed in plankton image analysis [99].

For plankton studies, the combination of quantitative imaging systems, autonomous platforms, and deep learning has fundamentally transformed our ability to observe pelagic ecosystems at ecologically relevant spatial and temporal scales [91,105]. In benthic environments, similar approaches are rapidly advancing habitat mapping, biodiversity assessment, and long-term monitoring, although challenges related to scene complexity and annotation effort remain.

Despite substantial progress, achieving robust, transferable, and fully automated counting across diverse aquatic environments remains an open challenge. Future developments are likely to emphasize standardized datasets and benchmarks, open image repositories and shared infrastructures, self-supervised and weakly supervised learning to reduce annotation costs, and tighter integration of imaging with complementary sensing modalities. Across both planktonic and benthic habitats, automated image analysis increasingly converges on a shared methodological framework—detection, segmentation, and regression—while remaining constrained by habitat-specific optical and ecological characteristics.

### 2.3. Image Processing in Forest Stand Mapping and Characterization

**Figure 3:**
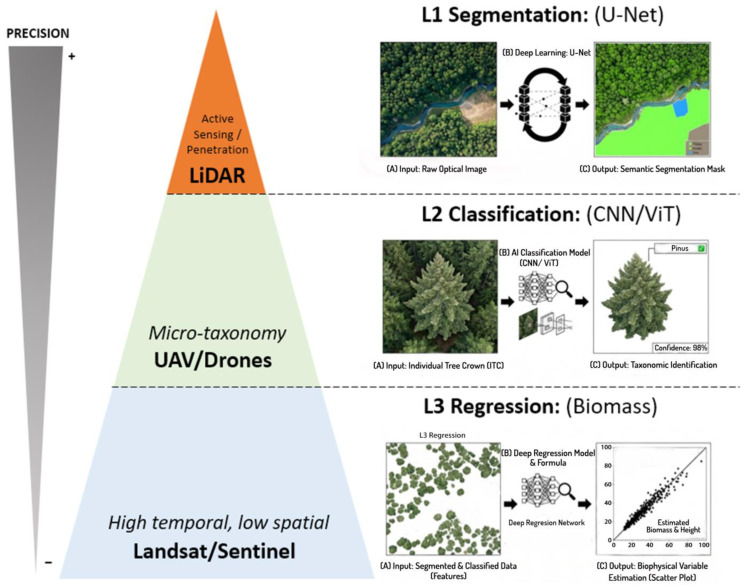
Hierarchical framework of terrestrial remote sensing technologies organized by spatial precision and coverage. The pyramid structure delineates three monitoring levels: (L1) high-precision active sensing (LiDAR) utilizing U-Net for semantic segmentation; (L2) micro-taxonomy via UAVs employing CNNs or ViTs for species classification; and (L3) high-temporal satellite imagery (Landsat/Sentinel) applied to biophysical regression tasks. The panel demonstrates the trade-off between spatial resolution and coverage, linking specific sensor types to their corresponding deep learning architectures.

The management of forest ecosystems in the present era demands a degree of responsiveness and accuracy that conventional analog techniques are no longer able to deliver. Historically, statistical sampling methods and visual observation in the field were the techniques used for forest inventory. While these methods are accurate at the local level, they are neither effective nor cost-effective in addressing the global dynamics of biodiversity loss and climate change. Indeed, a paradigm shift is occurring as a result of the combination of artificial intelligence algorithms, particularly deep learning, and a hierarchy of remote sensing sensors [99]. This technological convergence has precipitated a transition from purely descriptive monitoring to quantitative and predictive monitoring. The optical image, or that which is interpretable by the human eye, is not the sole source of value in contemporary remote sensing, primarily due to limitations such as signal saturation in dense canopies or the inability to resolve vertical structures. The model, therefore, is grounded in the widely utilized concept of spectral signature and the three-dimensional spatial configuration of the forest. In this context, AI functions as a deep contextual interpreter, transforming raw data (point clouds and pixels) into verifiable biophysical variables. This facilitates a “digital audit” of nature with unparalleled precision [106,107,108] (Figure 3).

The present section is concerned with a comparative examination of contemporary scientific methodology, with the aim of assessing the performance and limitations of the entire value chain from the perspective of data collection through active and passive sensors to processing using artificial intelligence architectures and their practical applications.

#### 2.3.1. Approaches to Data Collection: The Hierarchy of Sensors

The contemporary forest monitoring system is structured in the form of a pyramid, ranging from information obtained at the orbital level—which prioritizes global coverage over structural detail—to the measurement of the physical structure of individual trees, with a precision ranging from centimeters to meters. The methodology employed in the development of this multilevel structure is predicated on three complementary acquisition methods that encompass everything from regional dynamics to the three-dimensional architecture of the forest, effectively resolving the inherent trade-off between spatial extent and geometric resolution.

- *Regional and global scale: Spectroscopy and satellite systems*

Satellite constellations that observe the Earth form the basis for systematic monitoring, with the Landsat (National Aeronautics and Space Administration, NASA) and Sentinel-2 (European Space Agency, ESA) programs representing its primary components. The advent of large data sets has necessitated a transition from local workstations to cloud computing, leveraging platforms such as Google Earth Engine. This facilitates the analysis of comprehensive time series at the global level, a scale unattainable by drone-based approaches [109].

The spectral resolution of satellites is the primary factor determining their diagnostic capabilities, as it is the key factor in determining the extent of the biological analysis possible. While visible bands (RGB) provide information about external pigmentation, it is infrared radiation that reveals the internal physiology of plants. Healthy plants exhibit high reflectance in the near-infrared (NIR) due to the absorption of red light for photosynthesis and concurrent light scattering through the cellular structure of the mesophyll. Nevertheless, the primary methodological innovation resides in the capacity of neural networks to leverage the abundance of information inherent in shortwave infrared (SWIR) [110]. In contradistinction to conventional indices, such as the NDVI, which rely on rudimentary arithmetic and frequently fail due to signal saturation within dense forest environments, artificial intelligence models employ the SWIR to assess intricate parameters. These include the structure of lignin and cellulose, as well as the water content. This methodology facilitates the determination of water stress and the distinction of species that exhibit similar visual signatures but different chemical compositions, effectively overcoming the saturation barrier associated with traditional indices [111].

Remote sensing, in addressing the spectral challenge, also engages with the historical interplay between temporal and spatial resolution. Satellite platforms such as Sentinel-2 offer a high revisit frequency (5 days), yet their resolution (10–20 m) is limited, impeding the individualization of trees, a critical limitation compared to UAV data. To surmount this resolution gap, super-resolution algorithms founded upon Generative Adversarial Networks (GANs) are employed. These architectures can combine the spatial sharpness of archival or commercial satellite images with Sentinel’s temporal frequency, creating plausible spatial details to produce dense time series that are ideal for monitoring fine phenology [112].

Finally, to address the criticism that purely data-driven models do not act as *black boxes* that just correlate patterns without physical causation, they are coupled with biological physics using Radiative Transfer Models (RTMs) such as PROSAIL or DART. The deterministic equations used in this study to simulate the interaction of solar radiation with the canopy are based on real variables such as leaf area index (LAI) and chlorophyll concentration (C_ab_). However, a major limitation is that inverting these models to determine biology from observable light is computationally demanding and mathematically ill-posed, necessitating the use of Artificial Neural Networks (ANNs) trained on millions of RTM simulations. The result is a *physically guided AI* (Hybrid retrieval methods) that converts the satellite signal into biophysical variables, with a level of generalizability and mathematical traceability **unattainable by** purely empirical methods [110,113].

- *Precision scale: Unmanned aerial vehicles (UAVs)*

While satellites offer a comprehensive observation of the “landscape”, UAVs, also known as drones, facilitate a more detailed examination at the level of the “individual”, effectively bridging the resolution gap between ground surveys and orbital imagery. In the domain of microtaxonomy and field validation, UAVs are recognized as pivotal instruments within the realm of computational taxonomy.

The use of multispectral sensors at low altitudes allows for the detection of minute phenotypic differences such as branching architecture or floral phenology that would otherwise go undetected from space due to pixel mixing. Similarly, Structure from Motion (SfM) techniques and digital photogrammetry approaches can be used to build centimetrically accurate 3D point clouds and Digital Surface Models (DSMs). According to Kattenborn et al. [114], these reconstructions are used as digital ‘ground truth’ to train lower-resolution satellite models, allowing for precise upscaling of local data to the landscape level.

- *Three-dimensional digitization: LiDAR*

LiDAR (Light Detection and Ranging) is widely regarded as the gold standard for structural characterization. In contradistinction to passive optical sensors (like cameras used in SfM), which are limited to the upper canopy surface, LiDAR is an active sensor that produces laser pulses and creates a very dense point cloud with coordinates (X, Y, Z), capable of penetrating gaps to map the understory.

The AI system performs 3D semantic segmentation by classifying each point as either *leaf*, *trunk*, *ground*, or *branch*. The processing of these point clouds involves the utilization of geometric deep learning architectures, including PointNet [115], specifically designed to process unstructured data formats where traditional grid-based CNNs fail. This process facilitates the digital eradication of vegetation, thereby unveiling the Digital Terrain Model (DTM) beneath the canopy and enabling the reconstruction of the vertical structure of the forest. This reconstruction is imperative for the calculation of timber volume. Globally, NASA’s Global Ecosystem Dynamics Investigation (GEDI) mission has expanded this capability, providing global vertical profiles of forest structure that are essential for calibrating biomass models based on optical imagery which tend to saturate in high-density forests [116].

#### 2.3.2. Processing Through Deep Learning: The Flow of Analysis in Hierarchy

The analysis of forest images using artificial intelligence is not a monolithic process, but rather a multilevel structure in which each phase adds an additional level of complexity, both structural and taxonomic. This scientific process converts raw data into actionable information at the ecological level through three stages of analysis: segmentation, classification, and regression. The operational hierarchy under discussion is predicated on a logic of sequential dependency, in which the first level (i.e., spatial segmentation) constitutes the essential foundation for the second level (i.e., taxonomic identification), and this in turn for the third level (i.e., quantitative estimation of biomass), meaning that the accuracy of the final biological audit is strictly bound by the performance of the initial geometric segmentation.

- *Level 1: Spatial segmentation*

The initial fundamental filter is to ascertain which units are of interest. In order to accomplish this objective, the contemporary literature delineates between two mathematical perspectives that, despite their complementary nature, address disparate issues and computational scales.

Semantic segmentation (macro approach): The query *What type of class does this pixel represent?* is addressed. The model employs architectures such as U-Net to categorize each pixel in the image into groups, for example, water, forest, and land. The practical implementation of this standard is based on its skip connections, which allow for the fusion of deep contextual data with subtle spatial details. Consequently, U-Net emerges as a highly effective standard for quantifying total areas and identifying large-scale deforestation [117]. However, the procedure is limited in its functionality in that it does not take individuals into account; rather, it conceives of the forest as a continuous entity, rendering it insufficient for precision inventories where individual tree density is required.

Instance segmentation (micro approach): The following response is to be given to the question, *Where does each tree begin and end?* Architectures such as Mask R-CNN [30] or specialized packages such as DeepForest [118] go a step further, as they not only perform classification but also detect individual objects and generate a unique binary mask for each canopy. This computing capability enables direct delineation of individual crowns (ITC), a procedure that automates critical inventory functions such as tree counting and determination of crown projection area (CPA), a key geometric parameter for allometric biomass formulas [118]. In contrast to the semantic approach, this method provides high biological fidelity but is computationally intensive and prone to occlusion errors in dense canopies.

- *Level 2: Species classification and high-precision taxonomy*

After isolating the tree crown (ROI), the next challenge is determining its biological identity. Achieving success in forests with high biodiversity requires the use of an appropriate neural architecture that is adapted to the complexity of the ecosystem in question.

Convolutional neural networks (CNNs): These have been the benchmarks for texture evaluation. These devices use local filters to identify hierarchical patterns such as edges, textures, and forms. Their use is common when attempting to distinguish functional groupings with opposing morphologies. Because of their ability to memorize complicated texture patterns, they are particularly adept in distinguishing functional groups with contrasting morphologies (e.g., *Quercus* and *Pinus*) [119]. However, a notable limitation is their restricted “receptive field”, which focuses on local features at the expense of understanding the global shape of the tree, often leading to misclassifications in species with similar textures but different branching structures.

Vision transformers (ViT): These technologies represent the forefront of innovation for addressing the locality bias of CNNs. ViTs utilize self-attention techniques to analyze images as a collection of interrelated patches, capturing long-range dependencies across the image. This architectural paradigm facilitates the comprehension of the tree’s overall structure and the long-term relationships between its components. In practice, ViTs have been shown to be more effective in dense and mixed stands, as they are able to differentiate species based on minimal architectural and spectral variations that tend to confuse traditional sensors due to spectral saturation [120]. Nevertheless, this superior context awareness typically requires significantly larger training datasets compared to CNNs to achieve convergence.

- *Level 3: Regression for biophysical parameters*

At this final stage, the function of artificial intelligence is to cease classification and instead operate as a *virtual sensor* to calculate continuous variables. In this capacity, it acquires knowledge regarding the nonlinear relationships between physical structure and radiometry, modeling complex interactions that traditional linear regressions fail to capture. The process outlined herein enables the derivation of three fundamental metrics of the digital forest inventory.

Canopy height (CHM): The capacity of deep learning models to calculate the maximum vertical height (Z_max_) from stereo or monoscopic optical images is well-documented. This capability is developed through training with LiDAR data, which enables the inference of depth through textures and shadows. This inference is of crucial significance, given that height is the strongest predictor of above-ground biomass, as demonstrated by Lang et al. [108]. However, it is important to note that monoscopic height estimation remains an ill-posed problem compared to direct LiDAR measurement, often requiring domain adaptation techniques to generalize across different forest types.

Leaf area index (LAI): The model estimates the total leaf area per unit of soil (m^2^/m^2^). Deep spectral regression has been shown to be advantageous in that it circumvents the saturation phenomenon inherent to linear vegetation indices (like NDVI) in forests with high leaf area index (LAI), thereby providing a direct measurement of photosynthetic potential [121].

The basal area (G) is measured in square millimeters. AI, through the utilization of inverse allometry, ascertains a relationship between trunk diameter and segmented crown diameter (obtained in Level 1), subsequently adjusting projections based on intraspecific competition identified in the image [122]. A persistent challenge in this metric is the high variance in crown-to-trunk allometry in crowded stands, where simple geometric models may underestimate biomass without site-specific calibration.

#### 2.3.3. Essential Applications: Carbon, Health, and Resilience in the Age of Artificial Intelligence

The integration of advanced deep learning architectures with multispectral remote sensing and LiDAR facilitates the resolution of pivotal challenges in global environmental management. From simple observation tools to complex intelligence systems, these technologies have developed to provide unmatched temporal and geographical resolution in ecosystem dynamics. A synthesis of the key studies, sensor modalities, and AI architectures discussed in this section is presented in Table 5.

- *Carbon revolution: GEDI-optics merger and digital twins*

The integration of multimodal data has enabled the overcoming of the historical limitations of traditional forest inventories, thus leading to a qualitative advance in the quantification of above-ground biomass (AGB) and overcoming the saturation limits of optical-only models. The Global Ecosystem Dynamics Investigation (GEDI) mission, conducted by the NASA, is pivotal to this revolutionary endeavor. Despite the fact that it provides vertical profiles of forest structure with high-fidelity accuracy, its coverage is patchy due to the fact that it is based on discrete footprints.

In order to surmount this spatial discontinuity, the most recent methodological strategy is to utilize these GEDI footprints as ground truth labels in order to train deep learning models. These models process continuous images from optical satellites such as Landsat and Sentinel-2, achieving robust spatial extrapolation that neither sensor could achieve individually. The integration of the vertical accuracy of LiDAR footprints with dense optical time series facilitates the generation of Canopy Height Models (CHM) that exhibit a wall-to-wall resolution (continuous coverage) of 10 meters. The work of Lang et al. [108] represents a significant milestone in this field. In their study, the researchers trained a deep neural network with billions of GEDI footprints to produce the first global canopy height map. The error (RMSE) was reported to be approximately 6 meters, even in areas not covered by the original sensor.

Concurrently, this combination of sensors confronts an additional fundamental challenge: the mitigation of optical signal saturation, a prevalent issue in forests characterized by high biomass (exceeding 150 Mg/ha) where spectral indices lose sensitivity. This effect has been mitigated through the introduction of novel methodologies that combine topographic and synthetic aperture radar (Sentinel-1 SAR) data with Sentinel-2, which are processed using advanced algorithms such as CNNs or LightGBM. As recently demonstrated by Xu et al. [123] in the subtropical forests of China, the incorporation of SAR textures facilitates the circumvention of the conventional saturation threshold—as radar backscatter interacts with branch structure rather than just leaf pigmentation—thereby enabling the successful estimation of biomass through the extrapolation of LiDAR samples to the entire continuous surface.

The concept of Forest Digital Twins is predicated on the capacity for constant and accurate monitoring, moving beyond static maps to dynamic systems. This data is utilized by eminent institutions such as VTT in Finland within the framework of the VTT Technical Research Centre of Finland (2024) Forest Digital Twin Component (Forest DTC) for the Destination Earth project of the European Space Agency. The system is capable of developing virtual simulations of forests, with these simulations updated in real time. The significance of these twins lies in their ability to not only document the present situation, but also to facilitate the visualization of future projections in terms of growth and carbon capture. This is imperative to ensure transparency and monitoring in the certification of superior quality carbon credits, providing a verifiable audit trail that manual inventories cannot sustain.

- *Blue carbon: The boundary between wetlands and mangroves*

Due to their amphibious nature and difficult physical accessibility, the monitoring of blue carbon ecosystems (i.e., mangroves, marshes and seagrass beds) in situ has historically proven to be a challenging and costly endeavor. However, they play a pivotal role in mitigating climate change, as they store substantial amounts of carbon in both above-ground biomass and, particularly, in anoxic sediments. A global review of more than 400 studies demonstrates that remote sensing has established itself as the primary tool for analyzing the extent, dynamics, and carbon stocks of these ecosystems, with Landsat and Sentinel images being predominantly used for mangroves at regional and global scales [126].

A significant advancement has been the analysis of historical evolution using dense time series. Recent studies synthesized by Pham et al. [127] document the use of multi-decadal Landsat series to map changes in mangroves in Asia, Africa, and Latin America. Change detection and temporal segmentation algorithms are utilized to identify canopy loss, degradation, and recovery [127]. These approaches facilitate the differentiation between natural regeneration and active restoration trajectories in mangrove forests dominated by genera such as *Rhizophora*, *Avicennia*, and *Sonneratia*, dynamic patterns that remain indistinguishable in static or single-date assessments.

Beyond the canopy, the largest reservoir of blue carbon is found in the soil, which has driven the development of advanced predictive models. Maxwell et al. [128] generated a global map of soil organic carbon in mangroves at 30 m resolution. This map was created by integrating thousands of soil samples with climatic, topographic, and plant productivity variables derived from remote sensing. These variables were analyzed using ensemble machine learning models. This work demonstrates the potential to infer the carbon storage capacity of sediment from information observable from space, despite the fact that optical sensors do not directly detect the microbial processes responsible for such storage, relying instead on surface proxies to model underground conditions.

A recent systematic review has revealed that the combination of multispectral remote sensing (Landsat, Sentinel-2), SAR radar, and machine learning models is currently the most robust approach for estimating blue carbon stocks in mangroves. This approach allows estimates to be scaled from local plots to regional assessments relevant for the management and planning of restoration projects [129], while effectively mitigating the limitations of optical sensors in cloud-prone tropical regions where persistent cover frequently obstructs passive observation.

- *Predictive epidemiology in forests: From diagnosis to prognosis*

The use of remote sensing methods to identify forest pests and illnesses has significantly improved in recent years. While early methods focused on identifying trees that showed obvious evidence of damage or death, modern methods have made it possible to identify early signs of physiological stress that arise before symptoms become apparent. The application of preventive forest management strategies is made possible by this discovery. This change has been made possible by the combination of advanced machine learning models and time series spectral data, which capture temporal anomalies invisible to single-date inspections.

In this regard, a number of analyses have shown that spectral characteristics derived from multispectral photos contain information that can be used to predict future tree mortality. For instance, in 38 Canadian forest stands dominated by *Picea glauca*, *Populus tremuloides*, and *Pinus contorta*, Bergmüller and Vanderwel [124] utilized multispectral images obtained via UAV. Utilizing machine learning models that have been trained with spectral indices associated with crown vigor, they have developed a methodology that facilitates the prediction of the probability of individual tree mortality several months in advance, prior to the manifestation of deterioration in the field, effectively outperforming traditional visual assessments which rely on advanced degradation.

Furthermore, significant advances have been made in the field of early identification of particular pests through the utilization of deep learning models and the employment of high-resolution images. A paradigmatic case is the study by Qin et al. [125], who utilized RGB and multispectral images obtained with UAVs to detect *Pinus* trees infected by *Bursaphelenchus xylophilus*, the pine wood nematode, in forests located in eastern China. Utilizing an architecture based on YOLOv5, the authors reported classification accuracies exceeding 95% within their specific test dataset. These results highlight the potential of deep learning models to assist in identifying infected trees, particularly in controlled studies where visual indications are in their nascent stages. However, a limitation of these spatial-centric architectures is their reliance on visible texture changes, which may appear later than internal chemical shifts.

The most advanced frontier in this field is the combination of hyperspectral images with deep neural networks, which enables the identification of asymptomatic stress linked to subtle physiological changes, addressing the spectral limitations of standard RGB sensors. Hyperspectral images, due to their ability to capture hundreds of narrow bands, have the capacity to record minute alterations in parameters such as leaf cell structure, chlorophyll fluorescence, and water content. Incorporating both spectral and spatial information simultaneously, Nagasubramanian et al. [130] demonstrated that a three-dimensional convolutional neural network (3D-CNN) model, when applied to hyperspectral data, possesses the capacity to discern between infected and healthy plants prior to the occurrence of perceptible color changes, although this comes at the cost of significantly higher computational requirements (“curse of dimensionality”) compared to 2D models.

In forest systems, this approach has been applied for the early detection of diseases such as pine wilt. In 2018, Kim et al. [131] analyzed the hyperspectral signatures of *Pinus* affected by pine root knot nematodes. The study demonstrated that certain near-infrared and shortwave bands allow infected trees to be identified at early stages, when damage is not yet detectable by conventional multispectral sensors due to their broad bandwidths. These results indicate that the integration of hyperspectral remote sensing and deep learning facilitates the anticipation of the emergence of pests and diseases, thereby providing a critical window of opportunity for intervention prior to widespread dissemination.

It is evident from the combined findings of the aforementioned studies that the detection of forest pests and diseases has undergone a transition from a reactive approach, which is predicated on the identification of visible symptoms, to a predictive approach. The latter is characterized by its capacity to discern early physiological stress and to estimate the risk of future mortality by means of analyzing complex spectral signatures and time series, although the scalability of these methods from local UAV plots to global satellite monitoring remains a technological hurdle.

- *Fire management 4.0: From ‘nowcasting’ to ‘forecasting’*

The management of forest fires has evolved from a reactive approach, centered on the active detection of fires, to a predictive strategy that anticipates risk by evaluating the condition of the fuel. In this scenario, Live Fuel Moisture Content (LFMC) is considered one of the most influential variables due to its direct role in fire spread and ignition [132], replacing traditional field sampling methods which, despite their accuracy, are spatially sparse and unable to provide real-time regional monitoring.

The advent of remote sensing has facilitated the continuous and explicit estimation of LFMC, thereby circumventing the constraints imposed by conventional physical models which often suffer from complex parameterization requirements. As demonstrated by Marino et al. [133], a notable illustration of this phenomenon is the calculation of leaf-level foliar mass consumption (LFMC) in Mediterranean shrubs subject to the influence of *Cistus ladanifer* in Spain. This calculation was achieved through a meticulous integration of Sentinel-2 and MODIS images, alongside the utilization of empirical models and radiative transfer simulations. The conclusion drawn therein is that spectral indices combined with meteorological variables accurately reflect the seasonal dynamics of live fuel. In an effort to reduce errors when compared to purely mechanistic approaches and to capture the spatial variability of fire danger, Cunill-Camprubí et al. [134] produced weekly LFMC maps at the regional level for the entire Mediterranean basin between 2001 and 2021. These maps were created using MODIS thermal and spectral data together with Random Forest models, allowing for the modeling of non-linear relationships that linear spectral indices fail to capture.

In recent times, there has been a shift in focus from nowcasting to medium-term LFMC forecasting, a development which is of critical importance for the purposes of strategic planning. In a similar vein, Miller et al. [135] implemented deep learning models based on time series, incorporating architectures such as temporal convolutional neural networks (TempCNN). The training of these models was conducted using MODIS reflectance data and meteorological variables within fire-prone ecosystems in North America and Australia. Their findings indicate that live fuel moisture content (LFMC) can be predicted up to three months in advance, retaining relevant predictive power even over long time horizons; this makes it possible to anticipate critical situations before the high-risk period begins, significantly outperforming traditional autoregressive models which tend to degrade rapidly over longer forecast windows.

Concurrently, recent hybrid methods integrate land surface models with machine learning procedures to optimize fuel condition assessment. Santos et al. [136] developed a hybrid framework that combines biophysical simulations from land surface models with machine learning algorithms to calculate LFMC. This approach resulted in a substantial decrease in error when compared with exclusively physical techniques, whilst concomitantly reinforcing the operational capacity of these systems with regard to preventive fire management by correcting the systematic biases inherent in the physical simulations.

- *Monitoring biodiversity and illegality*

Remote sensing continues to encounter significant challenges, particularly in the context of biodiversity monitoring and the identification of illicit activities such as artisanal mining and selective logging. These challenges are especially pronounced in tropical regions characterized by persistent cloud cover and minimal disturbances. Optical sensors have been found to have significant limitations in such cases, as they rely on passive solar illumination and clear skies; consequently, the alterations caused by illegal activities tend to be episodic and subtle, and can therefore be overlooked for extended periods of time. The capacity of optical sensors to detect illicit activities such as artisanal mining or selective logging remains constrained in tropical regions characterized by persistent cloud cover [137]. This limitation arises from the episodic and subtle nature of disturbances in such environments, which only become discernible after a considerable duration.

The utilization of synthetic aperture radar (SAR) has emerged as a pivotal solution to address these constraints, as it functions independently of cloud cover or sunlight due to its active microwave transmission. Specifically, Reiche et al. [138] found that Sentinel-1 time series enable the identification of early-stage forest degradation and illegal deforestation in tropical rainforests, such as those in the Amazon and Indonesia. The authors detected alterations linked to selective logging and peatland drainage by analyzing changes in radar backscatter and their temporal consistency, even when these were not visible in contemporary optical images due to atmospheric obstruction. The employment of deep learning architectures, including U-Net segmentation networks, which facilitate high-resolution spatial mapping of novel illegal openings within intricate forest ecosystems [139], has further substantiated this viewpoint.

In addition to the issue of direct deforestation, there is the challenge of identifying subtle forest degradation, particularly that associated with agricultural expansion under canopy. This phenomenon has deleterious consequences for both carbon storage and biodiversity. In this context, [140] conducted an analysis of coffee agroforestry systems in Ethiopia and East Africa, merging vegetation indices extracted from satellite images and biomass measurements obtained in the field. The findings of the study demonstrated a strong correlation between woody biomass and spectral metrics, including NDVI and EVI. This facilitated the determination of gradients of forest deterioration caused by increased agriculture without total canopy eradication. The auditing of progressive structural transformations in multifunctional forest landscapes, which are frequently not given consideration within the remit of traditional binary deforestation detection systems (which classify only as “forest” or “non-forest”), is a particularly salient aspect of this approach.

- *Key limitations and challenges*

Ground Truth Scarcity: High-quality, spatially explicit field data is rare and expensive to collect, creating a significant bottleneck for training and validating robust deep learning models across vast areas.

Signal Saturation: In dense forest ecosystems with high biomass, traditional optical indices tend to saturate, making it difficult to estimate structural parameters accurately without auxiliary active sensor data like LiDAR or SAR.

Domain Adaptation: AI models trained on specific forest biomes often struggle to generalize to new geographic regions or different species compositions without extensive retraining or transfer learning techniques.

## 3. Conclusions and Perspectives

The integration of deep learning and computer vision into biological monitoring has fundamentally transformed the field from manual, labor-intensive workflows to high-throughput, automated pipelines. This review demonstrates that modern architectures, particularly deep convolutional neural networks (DNNs) and one-stage detectors like YOLO, consistently achieve high accuracies (exceeding 95%) across diverse taxa, including insect pests, aquatic microorganisms, and forest communities. Furthermore, the transition toward transformer-based architectures and hybrid models has enhanced the ability to handle long-range spatial dependencies and complex, dense aggregations where traditional methods often fail.

Beyond simple quantification, these technologies have enabled a shift from reactive monitoring to predictive ecological management. In forest ecosystems, the combination of multi-sensor data (thermal, LiDAR, and hyperspectral) with temporal models now allows for the forecasting of fire risk and the early detection of asymptomatic physiological stress months before symptoms become visible. Similarly, the deployment of intelligent “smart traps” and IoT-enabled edge devices is facilitating real-time surveillance of invasive species and disease vectors in both agricultural and public health contexts.

Despite these advancements, several critical challenges persist that define the future perspectives of the field:

**Resolution and Occlusion**: Detecting minute organisms like thrips or individuals hidden under dense vegetation remains difficult, necessitating further development in multimodal imaging (e.g., NIR and thermal fusion) to “see through” leaf shading.

**Data Scarcity and Generalization**: The “ground truth bottleneck”—the high cost of expert-annotated data—limits model performance in new geographic regions. Future research must prioritize self-supervised learning (SSL) and domain adaptation to leverage large unlabeled datasets.

**Biological Interpretability**: In the future, as models become more complex, the use of explainable AI (XAI) approaches such as Local Interpretable Model-agnostic Explanations (LIME [141]) will be essential for maintaining transparency, reproducibility, and to ensure that automated counts are based on valid biological features rather than spurious background noise.

**Taxonomic Limits**: Cryptic species that are morphologically identical still require genomic or microscopic verification; thus, integrating visual AI with molecular data or high-resolution trait-based analysis remains a key frontier.

In conclusion, while automated counting has reached a high level of operational maturity, the next generation of tools must focus on enhancing model transparency, cross-biome generalization, and the seamless integration of citizen science to provide a truly global, real-time assessment of biodiversity.

## Figures and Tables

**Table 1:** Comparative summary of technologies used for automated biodiversity monitoring.

Technology	Description	Main Advantages	Ecological Applications	Limitations/Considerations	References
Camera traps	Motion-activated cameras for non-invasive wildlife monitoring	Non-invasive, discreet, cost-effective, large data volumes	Wildlife monitoring in terrestrial, freshwater, and marine ecosystems	Time-consuming manual image processing; limited scalability	[1,2,3]
Automated classification (DNNs)	Deep convolutional neural networks for image analysis	High accuracy, rapid large-scale processing	Automated species identification and behavior recognition	Requires large labeled datasets and high computational resources	[4,5,6]
Drones (UAVs)	Aerial platforms with RGB, thermal, multispectral and LiDAR sensors	High resolution, programmable flight paths, broad spatial coverage, low risk to researchers	Vertebrate monitoring, habitat mapping, environmental assessment	Potential disturbance to wildlife (noise, altitude, proximity)	[7,8,11,12,13]
Thermal cameras on UAVs	Detection based on temperature differences	Efficient discrimination of endothermic organisms, effective under low light or dense vegetation	Nocturnal detection, monitoring of mammals and birds	Sensitive to ambient thermal conditions	[9,10]
Remote sensing (general)	Observation of spatial, temporal and structural ecosystem patterns	Large-scale, simultaneous ecosystem assessment	Plant and animal biodiversity studies	Resolution depends on sensor type	[14]
High spatial resolution imagery	Pixels generally < 10 m	Detailed landscape and habitat analysis	Fragmentation, connectivity, large animal detection	High cost and data volume	[15,16]
Hyperspectral sensors	Hundreds of narrow spectral bands	Species and physiological state differentiation	Plant diversity, habitat quality assessment; habitat proxies for fauna	Complex data processing	[17,18]
Thermal remote sensing	Measurement of emitted infrared radiation	Habitat thermal suitability, nocturnal detection	Fire monitoring, animal detection, thermal stress assessment	Affected by atmospheric conditions	[19,20]
Small satellite constellations	High temporal revisit from nanosatellites/microsatellites	Rapid change detection	Phenology, disturbance monitoring, habitat change	Lower spatial resolution	[21,22]
LiDAR	Active laser scanning for 3D models	Structural habitat information	Biomass, habitat complexity, species distribution modeling	High cost, intensive processing	[23,24,25]

**Table 2:** Comparative analysis of architectural complexity and suitability for biological monitoring scenarios. Note that FLOPs (Floating Point Operations) indicate computational cost, inversely related to battery life in field devices.

Architecture	Type	Resources (RAM)	Complexity (GFLOPs)	Inference Speed (FPS) *	Ideal Deployment Scenario
YOLOv8 Nano	CNN (One-Stage)	~3.2 M	~8.7 G	~105 (Real-time)	Edge/IoT Trap (Battery powered, limited RAM).
MobileNetV3	CNN (Lightweight)	~5.4 M	~0.2 G	~130 (Real-time)	Mobile App (Citizen science, rapid classification).
ResNet-50	CNN (Backbone)	~25.6 M	~4.1 G	~45	Server/Desktop (General purpose batch processing).
DETR (ResNet50)	Transformer	~41.0 M	~86.0 G	~12 (Slow)	High-End Lab Server (Complex occlusion, dense clusters).
Swin-T	Transformer	~29.0 M	~4.5 G	~25	Cloud Processing (Fine-grained taxonomy).

* Inference speeds are approximate values based on standard GPU benchmarks (e.g., NVIDIA T4/V100) on COCO datasets. Actual performance varies by image resolution and hardware.

**Table 3:** Overview of key image databases and monitoring platforms for insect recognition.

Resource/Dataset Name	Taxa/Group	Dataset Size	Description	Source
IP102	Insect pests (significant section on Hemiptera)	>75,000	Large-scale benchmark for insect pest recognition.	https://www.kaggle.com/datasets/rtlmhjbn/ip02-dataset accessed on 1 February 2026
AMI Dataset (AMI-GBIF and AMI-Traps)	Moths, Beetles, Bees, Wasps, Heteroptera (true bugs)	2.5 M (AMI-GBIF); Millions (Heteroptera)	Industry standard for moth trap imagery; focuses on non-lethal, AI-based monitoring.	https://www.insectmonitoring.org/ accessed on 1 February 2026
The Schmetterlinge Österreichs Dataset	Lepidoptera	>540,000	World’s largest dataset for Lepidoptera; expert-verified.	https://eurocc-austria.at/en/news/butterflies-and-conservation accessed on 1 February 2026
Butterfly and Moths	Butterflies and Moths	>12,000	High-quality, curated dataset designed for training classification models like EfficientNet.	https://www.kaggle.com/code/andytingzhiwei/butterfly-and-moth-classification-vit accessed on 1 February 2026
Beetle Byte Quintet	5 specific beetle species (e.g., grain beetles, weevils)	Not reported	Specialized dataset for species impacting stored products.	https://catalog.data.gov/dataset/data-and-code-from-ai-based-image-profiling-and-detection-for-the-beetle-byte-quintet-usin accessed on 1 February 2026
Roboflow Beetle Universe	Beetles and other crop pests	7200 (Pest Detection subset)	Open-source collection; includes “Pest Detection” and “Beetle Recognition Model” for object detection.	https://universe.roboflow.com/pest-classifier/beetle-2kes2 accessed on 1 February 2026

**Table 4:** Summary of the primary insect groups targeted by automated recognition methods.

Application	Model Architecture	Dataset Source	Main Result	Reference
Mosquitoes (Invasive species)	Multitiered CNN ensemble	Not reported	Distinguishes known from unknown species for monitoring.	[48]
Mosquitoes	VGG-16, ResNet-50, YOLOv5	Not reported	Achieved 97–98% accuracy in identification.	[49,50]
Olive fruit fly	Random Forest, SVM (IoT)	Not reported	94.5% accuracy using Raspberry Pi for in situ monitoring.	[51]
Fruit flies	Landmark & edge geometries	48 species	99% accuracy achieved at the genus level.	[52]
Aphids	VFNet, GFLV2, PAA, ATSS	Thousands of images	Stable precision; performance improved by merging nearby clusters.	[46]
Small sap-suckers	YOLOv8 + BEAM	Not reported	95.4% accuracy in real-field conditions focusing on tiny targets.	[53]
*Acyrthosiphon pisum*	Modified YOLOv8	Not reported	95.9% precision distinguishing nymphs from adults.	[54]
Psyllids	Multimodal (RGB + NIR + Thermal)	Not reported	95.3% counting accuracy; overcomes occlusion by leaf shading.	[47]
Moths	EfficientNet-B0	25 species	97.9% accuracy; highlights effectiveness of lightweight architectures.	[55]
Butterflies	ResNet50, EfficientNetB0, ButterflyNet	100 species	Successful identification at species level.	[56]
Lepidoptera	IoT + Smart Trap + DLMultiple	Multiple trap captures	97% detection accuracy; enabled generation of infestation maps (maize crop pests).	[57]
Beetles	CNNs (Transfer Learning)	14 families	96% accuracy including Curculionidae and Carabidae.	[58]
Beetles	Grounding DINO + Mask2Former	Large-scale data	97.8% accuracy processing laboratory tray data.	[59]
Maize weevils	MobileNetV2 (X-ray imagery)	Not reported	Detected internal infestation inside kernels (invisible to RGB).	[60]
*Leptinotarsa decemlineata*	YOLO (UAV imagery)	Not reported	99.8% training accuracy directly in the field.	[61]
Bees	EfficientNetV2L	Wing image dataset	98.1% accuracy; wing venation proved more stable than body images.	[62]
Bees	MobileNet-V2	Unspecified field-captured individuals	98.4% accuracy; identified as the most efficient among benchmarks.	[63]
Ants	YOLOv8 (with image slicing)	High density samples	~88% precision in crowded scenes using patch processing.	[64]
Ants	Multi-object tracking (MOT)	Foraging individuals	Improved tracking accuracy (HOTA) in overlapping scenarios.	[65]
Tiny Wasps	Grounding DINO + specialized CNNs	Not reported	Tiny specimens (1.2–4.5 mm). 96% accuracy at the genus level.	[66]

**Table 5:** Overview of primary image processing methods applied to forest.

Application	Model Architecture	Dataset Source	Main Result	Reference
Global Monitoring (Paradigm Shift)	Deep Learning (Contextual Interpreter)	Raw data (point clouds and pixels) from global sensors	Transition from descriptive to quantitative/predictive monitoring via technological convergence.	[106]
“Digital Audit” of Nature	AI as Deep Contextual Interpreter	Point clouds & Pixels	Transformation of raw data into verifiable biophysical variables with unparalleled precision.	[107,108]
Plant Physiology & Composition	Neural Networks (SWIR bands)	Spectral Data (SWIR bands) avoiding NDVI saturation	Assessment of lignin, cellulose, and water content; circumvents saturation in dense forests.	[110]
Species Differentiation (Chemical)	SWIR-based Analysis	Vegetation	Distinction of species with similar visual signatures but different chemical compositions (e.g., water stress).	[111]
Phenology & Fine Scale	Super-resolution GANs	Sentinel-2 time series fused with commercial imagery	Generation of dense time series with plausible “hallucinated” spatial details.	[112]
Biophysical Variables (C_ab_ LAI)	Hybrid Retrieval (RTM + ANNs)	Millions of RTM simulations (e.g., PROSAIL, DART)	“Physically guided AI” providing mathematical traceability and avoiding the “black box” effect.	[110,113]
Digital “Ground Truth”	SfM + Digital Photogrammetry	UAV (Drone) imagery with centimetric precision	Use of 3D reconstructions as ground truth to train and scale lower-resolution satellite models.	[114]
3D Structural Characterization	PointNet (Geometric Deep Learning)	LiDAR point clouds (Dense X,Y,Z data)	3D semantic segmentation (leaf, trunk, ground) and extraction of DTM beneath the canopy.	[115]
Land Cover/Deforestation (Macro)	U-Net (Semantic Segmentation)	Large-scale pixel data/Forest as continuous entity	Quantification of total areas; limited by viewing the forest as a continuous entity without individualization.	[117]
Individual Tree Inventory (Micro)	Mask R-CNN/DeepForest	Individual Tree Crowns (ITC) and binary masks	Crown delineation, automatic counting, and calculation of Crown Projection Area (CPA).	[30,118]
Functional Groups (Quercus vs Pinus)	CNNs (Texture Analysis)	High biodiversity forests with complex texture patterns	Differentiation based on contrasting morphologies and hierarchical texture patterns.	[114]
Complex Species Differentiation	Vision Transformers (ViT)	Dense/Mixed stands with minimal spectral variation	Effective in complex scenarios by analyzing interrelated patches (global attention).	[120]
Canopy Height (CHM)	Deep Neural Network	Billions of GEDI footprints (Global coverage)	First global canopy height map (RMSE 6 m) trained with GEDI footprints.	[108]
Leaf Area Index (LAI)	Deep Spectral Regression	High LAI Forests	Direct measurement of photosynthetic potential, avoiding saturation of traditional indices.	[121]
Basal Area (G)	Inverse Allometry	Segmented Crowns	Relationship between trunk diameter and crown diameter adjusted for intraspecific competition.	[122]
High Biomass Estimation (>150 Mg/ha)	Fusion: CNN/LightGBM	Optical + SAR textures (Subtropical forests)	Overcoming optical saturation limits by incorporating radar textures (Sentinel-1).	[123]
Mortality Prediction	Machine Learning (Spectral Indices)	38 Canadian forest stands (UAV multispectral data)	Prediction of individual tree mortality months before visual symptoms appear.	[124]
Pest Detection (Bursaphelenchus)	YOLOv5	RGB and multispectral UAV images (Eastern China)	>95% accuracy in detecting infected trees at early stages.	[125]

## Data Availability

No new data were created or analyzed in this study.
